# Health systems modelling – demonstrating the potential impact of diagnostic and treatment integration of human African trypanosomiasis using different health system structures

**DOI:** 10.1186/1472-6963-14-S2-P122

**Published:** 2014-07-07

**Authors:** Claudette S Sutherland, Chris Stone, Fabrizio Tediosi

**Affiliations:** 1 Department of Epidemiology and Public Health, Swiss Tropical and Public Health Institute, Basel, Switzerland; 2 University of Basel, Basel, Switzerland

## Background

Human African Trypanosomiasis (HAT) is a Neglected Tropical Disease (NTD) targeted for elimination. The declining prevalence of infection will change the demands on health systems to effectively detect cases. Detection of HAT currently relies on vertical surveillance programs where patients are identified in their villages and then required to travel long distances to HAT treatment centres (HTC). New diagnostics and interventions could change the future of service delivery of case detection and treatment; as local services would reduce out-of-pocket (OOP) expenditures and the inconvenience of travelling long distances. It is proposed that the integration of programs into the local health centres (LHC) could be modelled to forecast outcomes related to service delivery, patient accessibility, time spent in the system and resources used with current and new interventions.

## Materials and methods

A discrete-event simulation (DES) health systems model has been developed using SIMUL8^®^. The model simulates patients’ movement through the health system within a specified area. Different health system structures of both integrated (e.g. inclusion of local health centres) and non-integrated (e.g. vertical surveillance programs) approaches were constructed in the model. Data from current and new diagnostic and treatments have been simulated through the model in order to measure the impact of switching from a non-integrated to integrated health system.

## Results

Preliminary results suggest that integrated systems with new technologies will increase accessibility, decrease patient wait times but also require additional costs for training and for improving health infrastructures at the local level.

## Conclusion

An integrated health system could lead to improvements in coverage of treatment and reducing inequity in access to HAT treatment. While the initial additional costs of these interventions could be offset by savings in OOP payments, affordability to health systems should be carefully assessed. The analysis shows that health systems’ modelling is an informative tool for investment decisions regarding an integrated approach.

